# Development and Evaluation of a Reconstitutable Dry Suspension Containing Isoniazid for Flexible Pediatric Dosing

**DOI:** 10.3390/pharmaceutics12030286

**Published:** 2020-03-23

**Authors:** Oluwatoyin A. Adeleke, Rose K. Hayeshi, Hajierah Davids

**Affiliations:** 1 Division of Pharmaceutical Sciences, School of Pharmacy, Sefako Makgatho Health Sciences University, Pretoria 0208, South Africa; 2 Council for Scientific and Industrial Research, Pretoria 0001, South Africa; 3 DST/NWU Preclinical Drug Development Platform, Faculty of Health Sciences, North-West University, Potchefstroom 2531, South Africa; rose.hayeshi@nwu.ac.za; 4 Department of Physiology, Nelson Mandela University, Port Elizabeth 6031, South Africa; Hajierah.Davids@mandela.ac.za

**Keywords:** pediatric drug delivery, tuberculosis, reconstitutable dry suspension, isoniazid, polymer-lipid, microparticulate, direct emulsification

## Abstract

Tuberculosis (TB) is a major cause of childhood death. Despite the startling statistics, it is neglected globally as evidenced by treatment and clinical care schemes, mostly extrapolated from studies in adults. The objective of this study was to formulate and evaluate a reconstitutable dry suspension (RDS) containing isoniazid, a first-line anti-tubercular agent used in the treatment and prevention of TB infection in both children and adults. The RDS formulation was prepared by direct dispersion emulsification of an aqueous-lipid particulate interphase coupled with lyophilization and dry milling. The RDS appeared as a cream-white free-flowing powder with a semi-crystalline and microparticulate nature. Isoniazid release was characterized with an initial burst up to 5 minutes followed by a cumulative release of 67.88% ± 1.88% (pH 1.2), 60.18% ± 3.33% (pH 6.8), and 49.36% ± 2.83% (pH 7.4) over 2 h. An extended release at pH 7.4 and 100% drug liberation was achieved within 300 min. The generated release profile best fitted the zero order kinetics (R^2^ = 0.976). RDS was re-dispersible and remained stable in the dried and reconstituted states over 4 months and 11 days respectively, under common storage conditions.

## 1. Introduction

Tuberculosis (TB) remains a major global health problem present in every country in the world, regardless of the availability of standard treatment guidelines [1,2]. It is the leading cause of death from a single infectious agent, ranking above the Human Immunodeficiency Virus (HIV) with about 10 million new active infections and 1.5–2 million fatalities annually [1,3,4,5]. TB is an airborne, highly contagious disease often spread by coughing and sneezing. It is caused by strains of bacteria known as *Mycobacterium tuberculosis* (Mtb), which primarily infects the lungs (pulmonary TB) and, occasionally, other body parts (extra-pulmonary TB) [1,5,6,7]. TB has been identified as a key cause of economic devastation, revolving poverty and illness that has entrapped families, societies, and even entire countries, with women, children, and HIV patients being the most vulnerable [2].

Tuberculosis is a major cause of childhood death. The World Health Organization (WHO) recently estimated that 10%–11% of the global population infected with TB are children, with about 233,000 childhood deaths each year [1,3,6]. The TB mortality rate is 70% higher in children under the age of five than it is in adults in high burden areas [1,8]. Research indicates that children serve as reservoirs for active TB infection later in life, evidenced by the fact that globally, about 67 million children under the age of 15 have latent TB [1,9]. Latent TB infection is known as a state of persistent immune responses to stimulation by Mtb antigens with no evidence of clinical manifestations associated with active infection or symptoms of illness. Nevertheless, latent TB can develop into full-blown, active infection later [1].

Thus far, the greatest challenge to the successful treatment of TB in children is the significant shortage of efficient pediatric pharmaceutical formulations [1,10,11,12]. Despite the alarming statistics reported on the number of active TB cases in children, it is indeed shocking to note that to date, childhood TB has been generally neglected worldwide, evidenced by treatment and clinical care schemes mostly extrapolated from studies in adults [1,13,14,15]. Over the years, children have been largely excluded from clinical trials resulting in weak evidence-based treatment of pediatric TB infection. With the shortage of suitable child-friendly anti-TB pharmaceutical formulations, it is common global practice to split adult fixed-dose combination (FDC) preparations: (i) into fractions; (ii) crushed to be taken with food, milk, and other liquids; or (iii) extemporaneous compounding to allow for easy use as needed per child. These practices can lead to dose inaccuracies, reduced active drug potencies, impaired dosage stability, irregular bioavailability, and poor compliance [14,15,16]. Consequently, there is an urgent need for innovative treatment strategies that can contribute towards combating the TB epidemic in pediatric patients.

Isoniazid is the most widely used first-line anti-tubercular agent for the treatment and prevention of TB infection in both children and adults. It is a drug of choice as it is bactericidal, easily administered, inexpensive, and relatively non-toxic in children. Isoniazid is almost completely absorbed from the gastrointestinal tract and penetrates all body fluid cavities, in which drug levels are similar to serum levels [12,15]. Although subject to considerable hepatic metabolism (or first-pass effect) after oral dosing, it reaches concentrations well above the minimum inhibitory levels of Mtb in most tissues and TB lesions when given in standard doses [17]. Isoniazid is considered a class III drug according to the Biopharmaceutics Classification System (BCS) [18], meaning that it is highly hydrophilic (aqueous solubility = 125 mg/mL at 25 °C) [18,19] but not very permeable (log P = −0.64 at 25 °C) [19]. It is weakly basic, crystalline in nature, and does not display polymorphism [20]. The aim of this study was, therefore, to develop and characterize a microparticulate reconstitutable dry suspension containing isoniazid as a model drug. To date, there is not much detailed in literature on the use of anti-tubercular micro-suspensions for pediatric TB treatment or prophylaxis. Conventional dry suspensions are powder mixtures that require the addition of water at the time of dispensing. They are often widely acceptable, intended for oral administration, and usually choice alternatives when drug stability is a major concern. Dry suspensions are easy to use by any age group (particularly children) and, therefore, enhance patient compliance [13,21]. In this study, microparticulate dry suspensions as micro-structuring remains an ideal way of manufacturing highly efficient, rate-modulated pharmaceutical formulations that are beneficial to patients. Generally, microparticulate drug carriers are known to have high stability with excellent drug loading capacities for hydrophobic and hydrophilic drug moieties, enhanced bioavailability, and decreased toxicity [22]. The reconstitutable dry suspension (RDS) was prepared by blending liquid and solid interphases of drug and excipient into a homogenous mix that was lyophilized and pulverized to produce an isoniazid-loaded free-flowing powdery formulation. Formulation evaluation involved zeta potential and polydispersity index analyses, particle sizing, drug loading, dissolution testing, thermal behavior, structural transitions, surface morphology, crystallinity determinations, cytotoxicity, hydro- and environmental- stability testing.

## 2. Materials and Methods

### 2.1. Materials

Polyethylene glycol, poly (vinyl alcohol) 87%–89% hydrolyzed, coconut oil, ethylcellulose, hydrochloric acid, anhydrous sodium phosphate dibasic, 3-(4,5-dimethylthiazol-2-yl)-2,5-diphenyltetrazolium bromide (MTT), Trypsin—EDTA, trypan blue, dimethylsulfoxide (DMSO), camptothecin, potassium dihydrogen phosphate monobasic buffer salt, isoniazid, and phosphate buffered saline powder were purchased from Sigma Chemical Company (St. Louis, MO, USA). D-fructose was obtained from Merck Chemicals (Darmstadt, Germany). The human breast cancer (MCF-7) cell line was purchased from American Type Culture Collection (ATCC, Manassas, VA, USA). Dulbecco’s Modified Eagle’s Medium (DMEM) and fetal bovine serum (FBS) were procured from BD Biosciences (San Jose, CA, USA). All other reagents utilized were of analytical grade and used as received from the supplier.

### 2.2. Formulation of the Reconstitutable Dry Suspension Employing a Direct Dispersion Emulsification Method

The RDS was prepared using a direct dispersion emulsification technique coupled with lyophilization and dry milling. This involved emulsification of the aqueous phase, which contained 2%^w^/_w_ polyethylene glycol, 9%^w^/_w_ poly (vinyl alcohol), 4%^w^/_w_ D-fructose, 10%^w^/_w_ isoniazid dispersed in water with the non-aqueous phase made up of 3%^w^/_w_ ethylcellulose in coconut oil under constant mechanical blending (Silverson Machines, Inc., East Longmeadow, MA, USA) at 6000 rpm over 5–10 min until a monophasic emulsion was produced. With D-fructose as a lyoprotectant and sweetener, the mono-phased emulsion was exposed to liquid nitrogen for 15 min until completely frozen. Thereafter, the frozen sample was lyophilized (Benchtop Pro Freeze Dryer, VirTis, SP Scientific, Stone Ridge, NY, USA) at a temperature of −60 ± 2 °C and pressure of 124 ± 2 mTorr over 96 h to produce a solid lyophilisate that was then dry milled using a laboratory scale grinder (Kinematica GMBH, Eschbach, Germany). The RDS was stored away in airtight, opaque containers until further testing. 

### 2.3. Formulation Yield

The formulation yield was calculated by relating the total formulation weight (actual yield) to the mass of the component excipients used in the preparation of the formulation (theoretical yield). The constituting excipient plus the model drug and produced RDS formulation were weighed as separate entities using a laboratory scale balance (Kern EG 620-3NM, Kern and Sohn, GmbH, Balingen, Germany). The percentage yield was calculated using Equation (1) below:

(1) $$\%\mathrm{Yield}\;=\frac{\mathrm{Actual}\;\mathrm{yield}\;\left(g\right)}{\mathrm{Theoretical}\;\mathrm{yield}\;\left(g\right)}\times 100$$

### 2.4. Physicochemical Characterization of the Reconstitutable Dry Suspension

#### 2.4.1. Particle Size, Polydispersity Index, and Zeta Potential 

Measurement of particle size (PS) and polydispersity index (PDI) was based on the principle of dynamic light scattering using the Nano series Zetasizer (Malvern Instruments Ltd, Malvern, UK). For each quantification, RDS samples were re-dispersed in deionized water, appropriately diluted, and sonicated (Table-type Supersonic Cleaner KQ118, Nanjing T-Bota Scietech Instruments and Equipment, Co, Ltd., Nanjing, China) for 15 min at 37 ± 0.5 °C. All measurements were performed as three independent replicates with 10 readings per sample at a measurement angle of 173° and temperature of 25 °C. Zeta potential (ZP) is an indicator of particle surface charge, which determines particle stability in dispersions [23,24]. It was computed based on the Smoluchowski equation [25] using the Nano series Zetasizer with RDS samples dispersed in deionized water and placed in disposable folded capillary zeta cells maintained at 25 °C. For statistical relevance, samples were measured in triplicate with 20 runs per measurement cycle. 

#### 2.4.2. Visualization of Surface Morphology

The surface microstructure of the RDS was observed under a JEOL Transmission Electron Microscope (TEM) (JEM-2100 LaB6 200 kV Transmission Electron Microscope, JEOL, MA, USA). About 1 mg of the test sample was dispersed in ethanol and spotted on a carbon coated copper grid. Ethanol was allowed to evaporate under atmospheric conditions prior to sample loading into the TEM viewing stage (JEOL JEM-2100 LaB6 200 kV Massachusetts, USA). The Gatan Digital Micrograph Software was used to facilitate sample viewing. 

#### 2.4.3. Thermal Analysis

The thermal behavior of the drug loaded RDS and pure isoniazid (reference) was characterized using a differential scanning calorimetry (DSC) machine equipped with a 50-position automatic sampler (Q2000 Differential Scanning Calorimeter TA Instruments, New Castle, DE, USA). Separate aluminum crucibles containing about 9 mg of each sample were analyzed under an inert nitrogen flow of 25 mL/minute and heating rate of 10 °C.min^−1^ while an empty crucible subjected to the same testing condition served as the reference. All measurements were performed in triplicate per sample under three heating cycles at a temperature range of 0–220 °C.

#### 2.4.4. Determination of Crystallinity

The crystalline nature of the RDS formulation and pure isoniazid were evaluated under ambient conditions (25 ± 2 °C) using an X-ray diffractometer equipped with the X’Pert PRO data collector software (PANalytical Inc. MA, USA). Each sample (about 3 mg) analysis was performed at a voltage and current of 45 kV and 40 mA, respectively, and a 2θ range of 5 to 90° using a continuous scan mode with a scan step size of 0.03.

#### 2.4.5. Structural Elucidation

The RDS formulation and pure isoniazid chemical backbone structural transitions were determined using the Fourier transform infrared (FTIR) spectrophotometric approach. The FTIR spectra of each test sample was generated on a Perkin Elmer Spectrum 100 Series FTIR Spectrophotometer coupled with Spectrum V 6.2.0 software (Beaconsfield, UK). Initially, blank background measurements were taken, and then, about 5 mg of each test sample was placed on a clean holder situated on the test stage of the machine for structural analysis that was recorded at a frequency range of 650–4000 cm^−1^, scan time = 32 scans and resolution of 4 cm^−1^.

### 2.5. Drug Loading Efficiency

To determine the amount of isoniazid loaded in the RDS formulation, 10 mg sample was placed in 100 mL phosphate buffered saline (pH 7.4) with continuous stirring over 4 h (Five-Position Hot Plate/Stirrer, Model 51450 series, Cole-Parmer, IL, USA), to ensure complete dissolution and release of all entrapped drug molecules. The resulting solution was then appropriately diluted with distilled water and passed through a 0.45 µm nylon membrane Corning^®^ syringe filter (Corning Incorporated, NY, USA). The actual drug content was analyzed using Ultraviolet (UV) Spectrophotometry (PerkinElmer Lambda 35, UV/Vis Spectrometer, Perkin Elmer, Singapore) at a maximum wavelength of absorption for isoniazid, λ_max_ = 262 nm. The percentage of isoniazid load in the RDS formulation was mathematically computed with reference to the originally added amount (Equation (2)). Tests were conducted as three replicate samples.

(2) $$\%\mathrm{Drug}\;\mathrm{loading}\;\mathrm{efficiency}\;=\frac{\mathrm{Actual}\;\mathrm{drug}\;\mathrm{amount}\;\left(g\right)}{\mathrm{Theoretical}\;\mathrm{drug}\;\mathrm{amount}\;\left(g\right)}\times 100$$

### 2.6. In Vitro Dissolution Studies

In vitro dissolution studies were performed on an RDS formulation sample size containing an equivalent of 100 mg isoniazid. The study was carried out using the dissolution tester (Ewreka GmbH, DT 820 series, Heusenstamm, Germany). The RDS formulation was contained in a gelatin capsule (CapsCanada, Ontario, Canada), which was placed in a basket holder attached to the dissolution tester stirring shaft (United States Pharmacopeia Apparatus 1). The basket was then immersed into separate dissolution jars containing 500 mL of either pH 7.4 or pH 6.8 or pH 1.2 pre-heated buffered solution maintained at 37 ± 0.5 °C with continuous rotation at 100 rpm. Samples (5 mL) were removed at the end of the 2-h time-point for quantification. Furthermore, at pH 7.4, drug loaded RDS formulation underwent an extended release testing up to a point when 100% isoniazid liberation was observed. Briefly, samples (5 mL) were removed at pre-determined time intervals and replaced with 5 mL freshly prepared preheated, buffered solution (pH 7.4, 37 ± 0.5 °C). All experiments were performed in triplicate under sink conditions and isolated samples were appropriately diluted, filtered using a 0.45 µm nylon membrane Corning^®^ syringe filter (Corning Incorporated, NY, USA) and analyzed spectrophotometrically (PerkinElmer Lambda 35, UV/Vis Spectrometer, Perkin Elmer, Singapore) at λ_max_ of 262, 263, and 267 nm for pH 7.4, 6.8, and 1.2 buffered solutions, respectively, to determine the amount of isoniazid contained in each sample per time against buffer blanks. The actual quantity of isoniazid released per unit time was calculated using the linear polynomial calibration equations as follows: (a) pH 7.4: *y = 39.38x*; *R^2^ = 0.98*; (b) pH 6.8: *y = 32.96x; R^2^ = 0.99,* and (c) pH 1.2: *y = 45.77x; R^2^ = 0.98* where *x* and *y,* respectively, represent concentration (mg/mL) and absorbance. Percentage cumulative drug release [26] was computed relative to the total amount of isoniazid present in each medium at the end of the 2-h period. To get an indication of the release kinetics of the RDS formulation, the pH 7.4 release profile was selected for further mathematical model fitting over an extended period when 100% drug release was achieved. Resulting data were further analyzed using the KientDS version 3.0 open source software, which aided the selection of the model of best fit, which was based on mathematical computations, such as the zero, first, and second order kinetics, Higuchi, Korsmeyer-Peppas, Michaelis-Menten and Weibull approaches [19,24,27].

### 2.7. Stability Evaluations

#### 2.7.1. Short-Term Stability Testing under Different Environmental Conditions

The physicochemical stability of the RDS formulation (500.0 mg ± 2 mg per test) was monitored under different storage conditions over four months. A set of samples were placed in an enclosed glass holder that was transferred into the stability tester (Labcon PSIE RH 40 Chamber Standard Incubator, Laboratory Marketing Services, Maraisburg, South Africa) fixed at 30 °C ± 2 °C and a relative humidity of 65% ± 3% adapted from the WHO stability testing scheme for pharmaceutical products containing well established drug substances [28]. Another sample group was stored in airtight, opaque glass vials under standard room conditions (temperature: 25 °C ± 5 °C and humidity: 55% ± 5%). The last sample set was stored in airtight, opaque glass vials and placed in the refrigerator (Sanya Labcool Pharmaceutical Refrigerator, MPR-720R, Sanyo Electrical Biomedical Co. Ltd, CA, USA) at 4 °C ± 1 °C. Formulation stability was monitored at 0, 1, and 4 month intervals for changes in particle size, polydispersity index, zeta potential, and drug content. Samples were also physically examined for any changes in physical appearance or color changes. All tests were conducted in triplicate.

#### 2.7.2. In Vitro Aqueous Stability Assessment 

The stability of the RDS formulation in aqueous solution (mimicking the re-constitution process) was evaluated. RDS samples (500 ± 2 mg) were placed in airtight, opaque containers and dispersed in 50 mL sterile deionized water. Hydrated samples were prepared in triplicate per test conditions. Test conditions included placement under ambient conditions (25 ± 5 °C), as well as in the refrigerator (4 ± 2 °C) for 11 days. Hydrated samples (2 mL) were collected from each test vessel for isoniazid content quantification at 0, 1, 5, and 11 days using UVspectrophotometry, as described earlier. Samples were manually agitated daily and before collection to ensure uniform re-dispersion. All tests were carried out in triplicate.

### 2.8. Preliminary Formulation Toxicity Assessment 

The human breast adenocarcinoma (MCF-7) cell line was used for preliminary evaluation of the RDS formulation biocompatibility. The sample was dissolved in double-distilled water to a final stock concentration of 2 mg/mL, filter-sterilized through a 0.22 µm Cameo acetate membrane filter (Millipore Co., Bedford, Massachusetts), and stored at 4 °C until used. The negative control was 1% DMSO while 100 μM camptothecin, a known chemotherapeutic agent, was used as the positive control. The MCF-7 cell line was routinely maintained in Dulbecco’s Modified Eagle’s Medium (DMEM) supplemented with 10% heat-inactivated fetal bovine serum (FBS), at 5% carbon dioxide and 37 °C ± 0.1 °C. Cells were seeded into 96-well plates at a final concentration of 15,000 cells/well. Test samples were added at final well concentrations of 100, 50, 25 µg/ml, and incubated for 24 h. Thereafter, the spent medium was replaced with 200 µL of 0.05 mg/mL 3-(4,5-dimethylthiazol-2-yl)-2,5-diphenyltetrazolium bromide) (MTT) solution and incubated at 37 °C ± 0.1 °C for 3 h. The MTT was then aspirated from the cells and replaced with 200 µL DMSO to dissolve the formazan crystals. The absorbance was read at a maximum wavelength of 540 nm against DMSO as a blank using a microtiter plate reader (BioTek Powerwave XS, Winooski, VT, USA). The absorbance readings obtained were used to compute the number of viable cells present in the media. All results are presented as the mean reading ± standard deviation, and the statistical significance of all experimental data was evaluated using the GraphPad Prism 7 software (GraphPad, San Diego, CA, USA) using a two-way Analysis of Variance (ANOVA). The number of viable cells in both test and control samples were determined using Equation (3), and all tests were conducted in triplicate.

(3) $$\%\mathrm{Cell}\;\mathrm{viability}\;=\frac{\mathrm{Number}\;\mathrm{of}\;\mathrm{viable}\;\mathrm{test}\;\mathrm{cells}}{\mathrm{Number}\;\mathrm{of}\;\mathrm{viable}\;\mathrm{control}\;\mathrm{cells}}\times 100$$

## 3. Results and Discussions

### 3.1. Formulation Synthesis and Yield

The isoniazid-loaded RDS formulation was developed using a combination of the direct dispersion emulsification technique, lyophilization, and dry milling. A cream-white, free flowing isoniazid loaded RDS powder with an average yield of 87.43% ± 0.13% was obtained.

### 3.2. Size, Polydispersity Index, and Zeta Potential Determination and Morphology 

**Figure 1:**
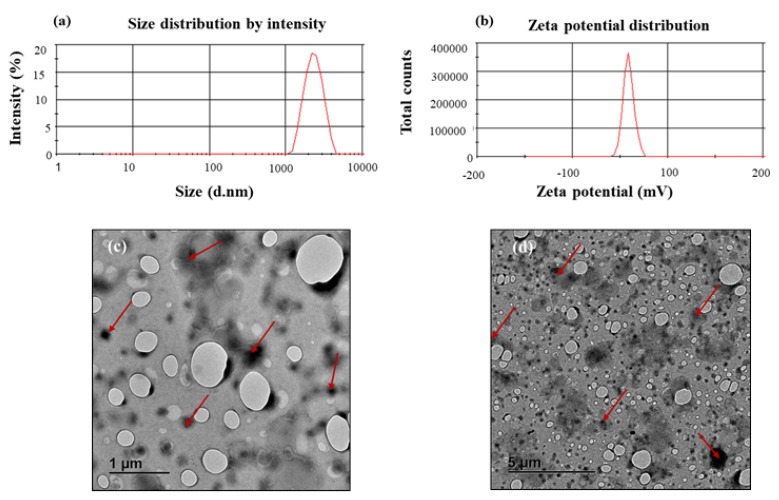
Representative graphs displaying: (**a**) particle size distribution, (**b**) zeta potential distribution, as well as TEM micrographs showing different surface topographies and characteristics of the reconstitutable dry suspension (RDS) particles at different scales: (**c**) 1μm and (**d**) 5 μm, respectively.

The RDS powder formulation showed an average particle size of 1.63 ± 0.20 µm, indicating its intrinsic micro-structure. The PDI can be described as a ratio that provides information about homogeneity of particle size distribution as it relates to a particular system serving as a useful reflection of the quality of the particulate system/dispersion ranging from 0.0–1.0 with values ≤0.1 relating to the highest quality of particulate dispersion, ≤0.3 as optimum, ≤0.5 as generally acceptable [24]. A generally acceptable PDI value of 0.37 ± 0.04 was recorded for the RDS powder, indicating that the particles were mostly homogenous and well dispersed within the formulation. The measured ZP was −41.10 ± 5.57 mV, showing a stable system, where a ZP value of ±30 mV is considered a stable and satisfactory formulation [24,29]. Representative graphs based on an independent measurement of the zeta potential and particle size distribution are shown in Figure 1a,b. Researchers have shown that TEM imaging is an effective and powerful tool for characterizing the morphology of nano- and micro-structured biomaterials and drug carriers as it uses more powerful beams to produce higher resolution images with more details and information [30,31]. The TEM micrographs showed minimally aggregated, dispersed RDS particles that appeared as darker areas relative to the background, with rounded outer morphologies (Figure 1c,d). 

### 3.3. Thermal Behavior 

**Figure 2:**
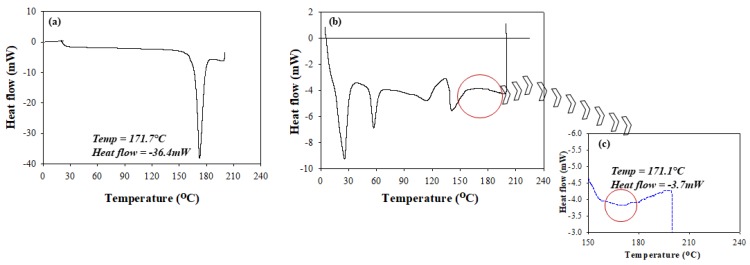
Differential scanning calorimetry (DSC) thermograms of (**a**) unformulated isoniazid, (**b**) RDS formulation, and (**c**) an expanded segment of the RDS thermograms showing the transitions that occurred with formulated isoniazid.

Salient changes in the thermal behavior of isoniazid relative to that of the RDS formulation were studied using conventional DSC methods. The recorded melting peak of pure, unformulated isoniazid was 171.7 °C, which compared well to literature [32]. The melting peak of isoniazid (Figure 2a) was characterized by its sharp and defined geometry confirming its purity and crystalline nature. The RDS thermogram was typified by the presence of multiple distinct sharp or broad peaks showing its physicochemically stable and multi-component state. The RDS formulation showed an intermittent appearance of sharp and blunt peaks representing its semi-crystalline nature (Figure 2b,c). The isoniazid melting peak identified on the RDS formulation thermogram (171.1 °C) was similar to that of the pure drug (171.7 °C), showing its stability within the excipient matrix. However, the formulated isoniazid peak presented as a broad band, which can be related to physical transitioning from crystalline into amorphous structures attributable to solvation and encapsulation of drug molecules into the processed polymeric carrier. A significant change in heat flow from −36.4 mW (unformulated isoniazid) to −3.7 mW (isoniazid in RDS) (Figure 2b,c) was seen, further supporting the likely drug encapsulation in the amorphous state, coupled with stable molecular dispersions within the polymeric chains [24]. Overall, the thermograms (Figure 2a–c) demonstrate the absence of any destructive/irreversible physicochemical interactions between isoniazid and the excipients during the RDS preparation process.

### 3.4. X-ray Diffractometry 

**Figure 3:**
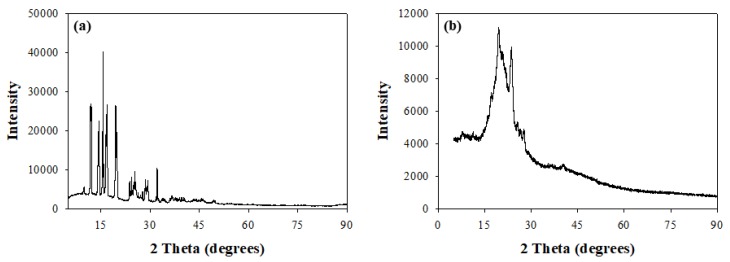
X-ray diffractograms of (**a**) unformulated isoniazid and (**b**) isoniazid loaded RDS formulation.

The changes in matrix crystallinity between pure isoniazid and the RDS formulation was further confirmed using X-ray diffraction analysis (XRD) with recorded diffractograms presented in Figure 3a,b. Diffractograms recorded for pure isoniazid showed high intensity, well-defined sharp peaks between 9.3θ and 32.3θ, with intensities as high as 39,885.7 validating the crystalline nature of isoniazid (Figure 3a). This trend differs from that observed for the RDS formulation, which presents broader, not so well-defined peaks between 13.9θ and 28.1θ at a maximum intensity of 11,142.9, which is much lower relative to the isoniazid diffractogram. Furthermore, low intensity (less than 4288.1) plateau-like, broad-banded sections were also identified between 5.3θ—14.6θ and 28.5θ—90.1θ, further confirming that the RDS formulation consists of more than one component, characterized with intermittent, mostly amorphous and minimal crystalline domains, i.e., a semi-crystalline nature (Figure 3b). These findings agree with the outcomes of the DSC analysis.

### 3.5. Structural Analysis

**Figure 4:**
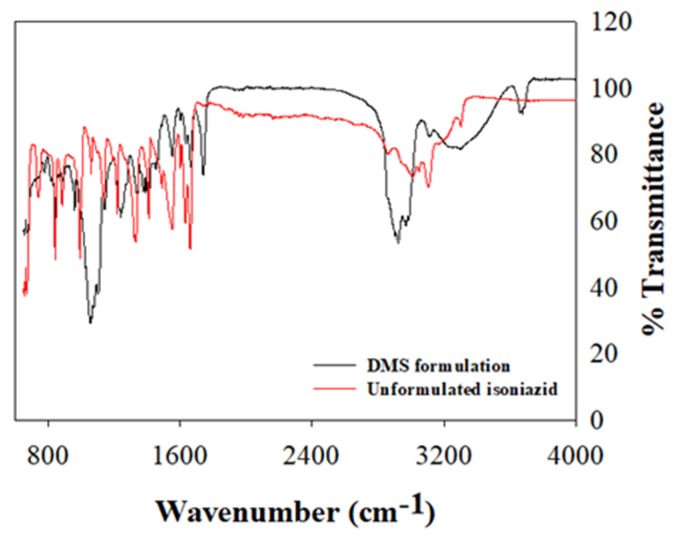
The Fourier transform infrared (FTIR) spectrum of the unformulated isoniazid and RDS formulation, showing different characteristic vibrational bands.

FTIR spectrum depicting characteristic vibrational frequencies of isoniazid in its unformulated state and encapsulated within the RDS formulation were compared (Figure 4). The vibrational band assignments for formulated and unformulated isoniazid were executed based on their positions, nature, magnitudes, and intensities relative to each characteristic functional group. The key bands plus wavenumbers identified and compared for both the RDS formulation and isoniazid in its pure state are presented in Table 1. The vibrational frequencies of key functional groups as it relates to the chemical backbone structure of isoniazid compares well for both pure drug and RDS formulation, with characteristic peaks presenting with varying intensities. The isoniazid loaded RDS formulation appeared overly dominant in most regions with some of its peaks suppressing those of pure isoniazid. This suggests physical solid–liquid/solid–solid dispersion and significant drug encapsulation. The O–H stretch recorded at 3676 cm^−1^ for the RDS formulation further supports the physically dispersed state in the water/oil medium, followed by snap-freezing and lyophilization. 

### 3.6. Drug Content 

The percentage content of isoniazid encapsulated within the RDS formulation matrix was computed relative to the theoretical drug content, totaling 94.12% ± 2.10%. This indicates that there was minimal drug loss during the RDS manufacturing and processing phases.

### 3.7. Drug Release Behavior

To understand the in vitro drug release behavior and kinetics, dissolution studies were carried out on the RDS formulation containing an equivalence of 100 mg isoniazid in pH 7.4, 6.8, and 1.2 buffered solution over two hours under biorelevant conditions. Percentage cumulative drug release (%CDR) was calculated as the total amount of isoniazid liberated from the RDS formulation matrix, with an increase or decrease in %CDR representing a respective rise or decline in the release rates. %CDR varied for each dissolution media (pH 1.2 = 67.88% ± 1.88%, pH 6.8 = 60.18% ± 3.33%, and pH 7.4 = 49.36% ± 2.83%). Isoniazid release decreased as media pH increased. In other words, a reduction in the pH of the aqueous micro-environment seemed to impact the processes of formulation wetting, pore formation and closure, water penetration, phase transitioning, drug-excipient dissolution and degradation, changes in drug-excipient physical interaction and solubility process, and eventual diffusion of drug molecules coupled with gradual matrix geometry transitions (erosion or swelling) [33]. The RDS formulation demonstrated the potential to stabilize and release the isoniazid molecules in different dissolution media with significant differences in the percentage of drug released under these different conditions. 

**Figure 5:**
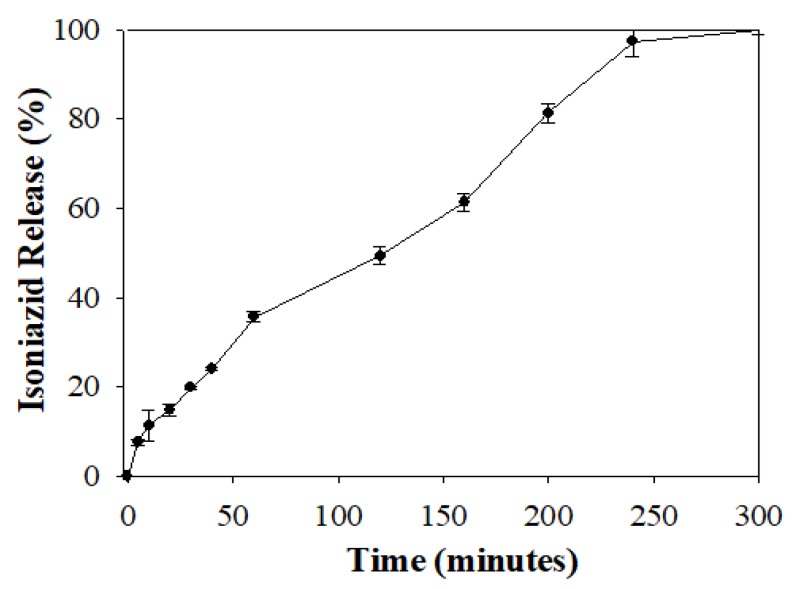
Graphical profile showing 100% isoniazid release from the RDS formulation over time.

In addition, isoniazid release at pH 7.4 was selected for further mathematical model fitting to understand the RDS formulation release kinetics over a period when 100% drug liberation was achieved (Figure 5). In vitro isoniazid release from the RDS formulation at pH 7.4 was characterized with an initial burst at 5 min (7.51 ± 0.72 %), followed by a relatively consistent increase in drug release over time, until complete release (100%) was recorded at approximately 300 min. 

The generated profile (Figure 5) was further analyzed using mathematical kinetic models employing the KinetDS, version 3.0 open source freeware. Release profile analysis and model of best fit selection was based on a combination of robust validation quantities, namely the correlation coefficient (R^2^) closest to one and lowest Akaike Information Criterion (AIC) numerical value (Table 2) [31]. On this basis, the zero order kinetic model provided the best fit parameters (R^2^ = 0.976 and AIC = 74.080) for the isoniazid release data depicted in Figure 5. This indicates that isoniazid release from the RDS formulation is consistent over time, irrespective of the initial drug concentration. In principle, zero order drug release mechanism is beneficial for achieving continuous drug plasma and biological fluid levels, reducing dosing frequency, and improving patient compliance, aiding desired pharmacotherapeutic efficacy [34]. 

### 3.8. Short-Term Formulation Stability Assessment 

#### 3.8.1. Stability Evaluation under Varying Storage Conditions

Evaluation of formulation stability was performed in triplicate per sample under multiple storage conditions: Stability tester—30 °C ± 2 °C and 65% ± 3%; Room—25 °C ± 5 °C and 55% ± 5%; and Refrigerator—4 °C ± 1 °C; over four months employing particle size, polydispersity index, zeta potential, and drug content as indicators of stability. Results showed minimal numerical differences in indicators measured, indicating that the isoniazid loaded RDS formulation can be described as stable under the prescribed environmental storage conditions (Table 3). A slight alteration in physical appearance relating to a color change from white to cream-white was observed at four months, under accelerated storage in the stability chamber (30 °C ± 2 °C; 65% ± 3%) and room conditions (25 °C ± 5 °C; 55% ± 5%). Despite the fact that stability indicators remain closely related, the slight color change is undesirable, considering patient acceptance, making these environments unsuitable for the storage of the RDS formulation. Therefore, the most ideal storage setting for the RDS formulation is, thus, at 4 ± 1 °C as stability indicators are comparable with no color change observed (Table 3).

#### 3.8.2. Formulation Stability in an Aqueous Environment

The stability of the drug loaded RDS formulation in an aqueous medium was investigated under select storage conditions (room and refrigeration) over 11 days, mimicking storage duration for commonly reconstituted antibiotic solutions/suspensions. Percentage drug content was chosen as the hydrostability indicator, and overall, there were insignificant changes in its numerical values under room or refrigerated storage conditions, with no visible color changes (Table 4). Summarily, the RDS formulation is stable in the aqueous medium over 11 days, either refrigerated or stored under ambient conditions, suggesting that the RDS formulation is a potentially useful preparation for re-constitution purposes, especially in pediatrics.

### 3.9. Cell Viability Assessment

**Figure 6:**
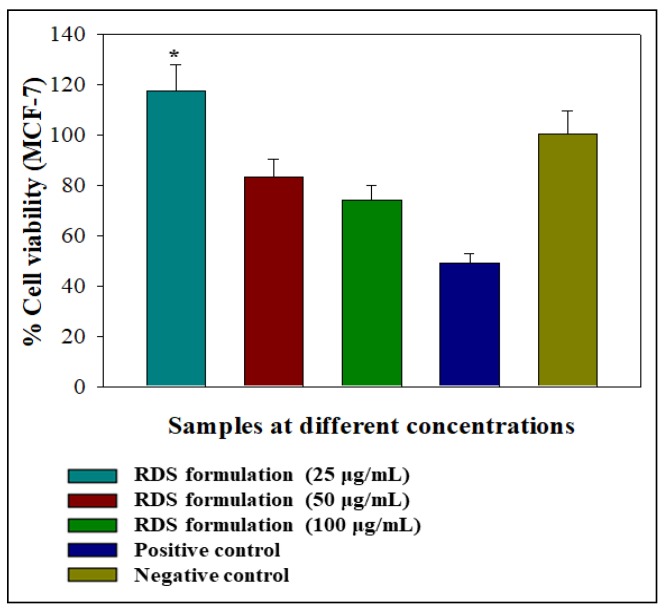
Graphical illustration of the percentage cell viability 24-h post-exposure to the RDS formulation in the human breast cancer (MCF-7) cell lines as a prototype. Statistical difference between two data sets was considered significant when *p < 0.05.*

Preliminary assessment of the effects of the RDS formulation on viability was performed on MCF-7 cell lines. Tests were conducted over 24 h using different concentrations of the RDS ranging from 0 μg/mL (negative control) to 100 μg/mL. This cell line is routinely used as a prototype for assessing the biocompatibility of drugs, delivery systems, and biologicals that are non-specific for treating carcinomas [35,36,37]. Graphical representations of the impact of different test formulation concentrations on cell viability relative to camptothecin (positive control) and the negative control are presented in Figure 6. The RDS lowest concentration (25 μg/mL) caused significant MCF-7 cell proliferation (*p* < 0.0500: *p = 0.0001*). The increased viability may signify that the RDS formulation supported cell growth at a low concentration (25 μg/mL) and can be considered an indication of biocompatibility. This may be attributed to the drug or excipient concentration or a combination of both. In contrast, the 50 and 100 μg/mL reduced cell viability, although not significantly (*p* > 0.0500: 50 μg/mL—*p = 0.0056*; 100 μg/mL—*p = 0.0001*). Nevertheless, higher concentrations of the RDS (50 and 100 μg/mL) did not reduce cell viability as much as the camptothecin that decreased it to 48.93% ± 3.99%. In general, the RDS was well-tolerated by the MCF-7 cells at all test concentrations. Cellular responses following exposure to the formulation can be described as biphasic and dose-dependent, a phenomenon associated with hormesis (a two-phased adaptive response of cells and organisms to increasing or decreasing amounts of external stress, e.g., drug, chemical substances, and disease state) [38,39]. It is not unusual for cells to exhibit hormetic effects as they are biological systems known to be dynamic and constantly evolving. These initial findings form a baseline for future work on understanding the biocompatibility of the RDS formulation and its components (active drug and excipients) in vitro and in vivo.

## 4. Conclusions and Future Work

In this study, isoniazid loaded reconstitutable dry suspension was prepared using the direct dispersion emulsification technique, coupled with lyophilization and dry milling. The direct dispersion technique produced a relatively high yield (87.43% ± 0.13%) of RDS particles with good drug loading capabilities (94.12% ± 2.10%). The RDS formulation showed no significant evidence of toxicity supported by outcomes from viability studies in the MCF-7 cells. The formulation was physicochemically stable, mostly amorphous, with marginal intermittent crystalline domains, and had no irreversible alterations in its backbone chemical structure. It demonstrated the ability to regulate isoniazid release in a controlled, zero order manner, and was environmentally stable under common storage conditions, either as a dry powder or in the hydrated form. The findings from this work may contribute towards improving flexible pediatric dosing for tuberculosis drug treatment, considering the current global shortage of such preparations, especially for the first-line anti-tubercular drugs. 

To establish a course for further investigations, we identified the need to extend biocompatibility testing for the RDS formulation and its components to normal cells and tissue isolates from animal models (e.g., mice, rabbit, or pigs) employing cytotoxicity assays and histopathological techniques, respectively. Further preclinical evaluation of pharmacokinetics, efficacy, and eventual optimization of isoniazid dosing and absorption from the RDS formulation, in animal models similar to humans, such as pigs, are important next steps. Considering the hydrophilic/hydrophobic and particulate nature of the RDS, we anticipate that it can find extensive use as an effective carrier for other anti-tubercular drugs (e.g., pyrazinamide, rifampicin) suitable for pediatrics irrespective of their solubilities and molecular weights. For implementation purposes, a range of these bioactives would need to be tested in vitro/in vivo and optimized to ensure desirable drug loading, controlled release, and absorption for the intended pharmacotherapeutic application. Overall, the RDS formulation reported herein has the potential for improving tuberculosis treatment within the pediatric population. 

## Figures and Tables

**Table 1:** FTIR vibrational frequencies representing formulated and unformulated isoniazid key functional groups.

Functional Groups and Remarks	Vibrational Frequencies (cm^−1^)	
	Unformulated Isoniazid	Isoniazid Loaded RDS Formulation
C–C–C–C and C–N–C–C torsion	653	665
NH_2_ rock	677	677
C–C–C out of plane bending	746	757
C–C–H out of plane bending	850 and 1022	850 and 1023
C–N–C and C–C–C in plane bending	1063	1062
Aromatic C–N stretching	1344	1330
C–C–H in plane bending	1210	1212
O=C-N in plane bending	1330	1333
C–N–H in plane bending	1411	1408
C–C stretching	1477	1471
C=O stretching	1558	1552
NH_2_ scissoring	1632	1635
Aromatic C–H stretching	3052	3054
N–H stretching	3307	3308
O–H stretching	-	3676

**Table 2:** Representative mathematical models and their respective fit parameters.

Mathematical Models	R^2^	AIC
Zero order	0.976	74.080
First order	0.159	176.881
Second order	0.091	129.633
Korsmeyer-Peppas	0.997	110.291
Weibull	0.990	92.616
Michaelis-Menten	1.000	120.737
Higuchi	-0.341	127.138

**Table 3:** Stability indicators measured under the different storage conditions.

RDS Formulation Test Conditions	Time (Months)	Stability Indicators				
**Stability tester**		**PDI*^a^***	**ZP*^b^* (mV)**	**PS*^c^* (µm)**	**DC*^d^* (%)**	**Color**
	0	0.37	−41.10	1.63	94.12	No change
	1	0.40	−42.59	1.58	94.07	No change
	4	0.39	−43.60	1.82	93.76	Slight change
**Room**	0	0.37	−41.10	1.63	94.12	No change
	1	0.44	−42.71	1.69	93.95	No change
	4	0.46	−40.51	1.97	91.05	Slight change
**Refrigerator**	0	0.37	−41.10	1.63	94.12	No change
	1	0.39	−41.65	1.61	93.95	No change
	4	0.38	−40.91	1.70	94.02	No change

**^a^** Particle Size (standard deviation ≤ 0.13 μm in all cases), **^b^** Polydispersity Index (standard deviation ≤ 0.03 in all cases), **^c^** Zeta Potential (standard deviation ≤ 1.03 mV in all cases), **^d^** Drug Content (standard deviation ≤ 0.99% in all cases).

**Table 4:** Hydrostability indicators at different time-points, under ambient and refrigerated conditions.

Test Conditions	Time-Points (Days)	Drug Content (%)	Discoloration
**Room/Ambient**	0	94.12	None
	1	93.18	None
	5	93.61	None
	11	92.89	None
**Refrigerator**	0	94.12	None
	1	93.33	None
	5	93.79	None
	11	92.56	None
